# Continuous Hemodialysis in Severe Lithium Intoxication: A Successful Case Management in a 67-Year-Old Woman

**DOI:** 10.7759/cureus.98769

**Published:** 2025-12-08

**Authors:** Masaatsu Kuwahara, Hideaki Imanaka

**Affiliations:** 1 Department of Emergency Medicine and Critical Care, Takarazuka City Hospital, Takarazuka, JPN

**Keywords:** continuous hemodialysis (chd), elderly patient, lithium intoxication, neurotoxicity, renal replacement therapy

## Abstract

Lithium toxicity is a potentially life-threatening condition characterized by neurological, gastrointestinal, and cardiovascular manifestations. Although intermittent hemodialysis (IHD) is the standard of care for severe lithium poisoning, continuous hemodialysis (CHD) or continuous renal replacement therapy (CRRT) may be preferred in cases of hemodynamic instability or severe neurological symptoms. We report a case of severe lithium intoxication in a 67-year-old woman successfully treated with CHD. Treatment was initiated before the serum lithium concentration became available. Despite marked neurological symptoms, including altered consciousness and tremors, CHD achieved gradual clinical improvement and full recovery without symptom recurrence.

## Introduction

Lithium toxicity was first described in 1898, and its clinical significance became more widely recognized in 1949 when lithium chloride was used as a sodium substitute in patients with heart failure [1]. After the introduction of routine serum monitoring in the 1970s, lithium carbonate received approval in the United States as a therapeutic agent for acute mania and bipolar disorder. Currently, approximately 6,000 to 7,000 cases of lithium intoxication are reported annually to US poison control centers [2]. Lithium carbonate remains a mainstay in the treatment of bipolar and mood disorders, but its narrow therapeutic index (0.6-1.2 mmol/L) increases the risk of toxicity due to overdose, renal impairment, or dehydration [3]. Severe lithium poisoning (serum lithium > 3.0 mmol/L) can lead to encephalopathy, tremors, seizures, and cardiac arrhythmias [4]. Although intermittent hemodialysis (IHD) is the standard treatment for lithium intoxication, continuous hemodialysis (CHD) or continuous renal replacement therapy (CRRT) may be preferable in patients with hemodynamic instability, aspiration risk, or severe neurological impairment [5]. We present a case of severe lithium intoxication in an elderly woman successfully managed with CHD, with treatment initiated before the serum lithium concentration became available, resulting in full neurological recovery.

## Case presentation

A 67-year-old woman with a medical history of type 2 diabetes, hypertension, and depression was brought to the emergency department after experiencing tremors, slurred speech, and recurrent vomiting for two days. Her home medications included lithium carbonate 300 mg/d, lamotrigine 100 mg/d, aripiprazole 3 mg/d, duloxetine hydrochloride 30 mg/d, flunitrazepam 1 mg/d, doxazosin mesylate 1 mg/d, nifedipine 20 mg/d, carvedilol 2.5 mg/d, tirzepatide 5 mg subcutaneously once weekly, telmisartan 80 mg/d, and trichlormethiazide 2 mg/d.

Upon arrival (day 0), she appeared drowsy with a Glasgow Coma Scale (GCS) score of 1-1-4 and was repeatedly vomiting [6]. Her blood pressure was 190/73 mmHg, and oxygen saturation was 95% while receiving high-flow nasal cannula (HFNC) oxygen at 40% FiO₂ and 40 L/min flow. Her temperature was 36.2°C, and the respiratory rate was 30 breaths/min, indicating tachypnea. Pupils were 3 mm bilaterally with brisk light reflexes. The skin showed no abnormal moisture or dryness. The laboratory test results are summarized in Table 1.

**Table 1:** Laboratory Findings on Arrival TP: total protein; ALB: albumin; T-Bil: total bilirubin; AST: aspartate aminotransferase; ALT: alanine aminotransferase; ALP (IFCC): alkaline phosphatase (International Federation of Clinical Chemistry); LD: lactate dehydrogenase; BUN: blood urea nitrogen; CRE: creatinine; Na: sodium; K: potassium; Cl: chloride; Ca: calcium; CK: creatine kinase; AMY: amylase; CRP: C-reactive protein; GLU: glucose; WBC: white blood cell count; RBC: red blood cell count; Hb: hemoglobin; Ht: hematocrit; PLT: platelet count; serum lithium: serum lithium concentration; PT: prothrombin time; APTT: activated partial thromboplastin time; pH: potential of hydrogen; pCO₂: partial pressure of carbon dioxide; HCO₃⁻: bicarbonate.

Parameter	Measured value	Reference range	Unit
TP	8.2	6.6–8.1	g/dL
ALB	4.3	4.1–5.1	g/dL
T-Bil	2.4	0.4–1.5	mg/dL
AST	14	13–30	U/L
ALT	10	7–23	U/L
ALP (IFCC)	135	38–113	U/L
LD	129	124–222	U/L
BUN	46.9	8–20	mg/dL
CRE	1.38	0.46–0.79	mg/dL
Na	140	138–145	mmol/L
K	4.2	3.6–4.8	mmol/L
Cl	106	101–108	mmol/L
Ca	10.6	8.8–10.1	mg/dL
CK	68	41–153	U/L
AMY	196	44–132	U/L
CRP	0.04	≤0.14	mg/dL
GLU	156	73–109	mg/dL
WBC	7.03	3.30–8.60	×10³/μL
RBC	3.87	3.86–4.92	×10⁶/μL
Hb	11.8	11.6–14.8	g/dL
Ht	37.6	35.1–44.4	%
PLT	185	158–348	×10³/μL
Serum lithium	3.1	0.3–1.2	mEq/L
PT	79	70–130	%
APTT	23.9	24–34	sec
pH	7.389	7.35-7.45	
pCO_2_	37.2	35-45	mmHg
HCO_3_⁻	22.5	21-28	mmol/L

Brain magnetic resonance imaging (MRI) and cerebrospinal fluid (CSF) analysis were negative for acute stroke or meningitis (Table 2).

**Table 2:** Cerebrospinal Fluid (CSF) PCR Test Results All cerebrospinal fluid PCR tests were negative, indicating no evidence of bacterial or viral meningitis/encephalitis.

Pathogen	Result	Reference (normal)
Meningitis/encephalitis nucleic acid panel	Negative	Negative
*Escherichia coli* K1	Negative	Negative
Haemophilus influenzae	Negative	Negative
Listeria monocytogenes	Negative	Negative
Neisseria meningitidis	Negative	Negative
Group B *Streptococcus*	Negative	Negative
Streptococcus pneumoniae	Negative	Negative
Cryptococcus neoformans/gattii	Negative	Negative
*Cytomegalovirus* (CMV)	Negative	Negative
Enterovirus	Negative	Negative
Herpes simplex virus type 1 (HSV-1)	Negative	Negative
Herpes simplex virus type 2 (HSV-2)	Negative	Negative
Human herpesvirus 6 (HHV-6)	Negative	Negative
Human parechovirus	Negative	Negative
Varicella-zoster virus (VZV)	Negative	Negative

**Figure 1:**
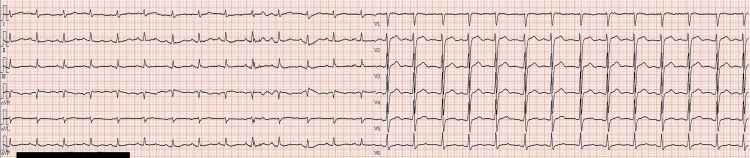
Electrocardiographic Findings on Arrival

A 12-lead electrocardiogram demonstrated a corrected QT interval (QTc) of 0.500 seconds, indicating QT prolongation (Figure 1).

**Figure 2:**
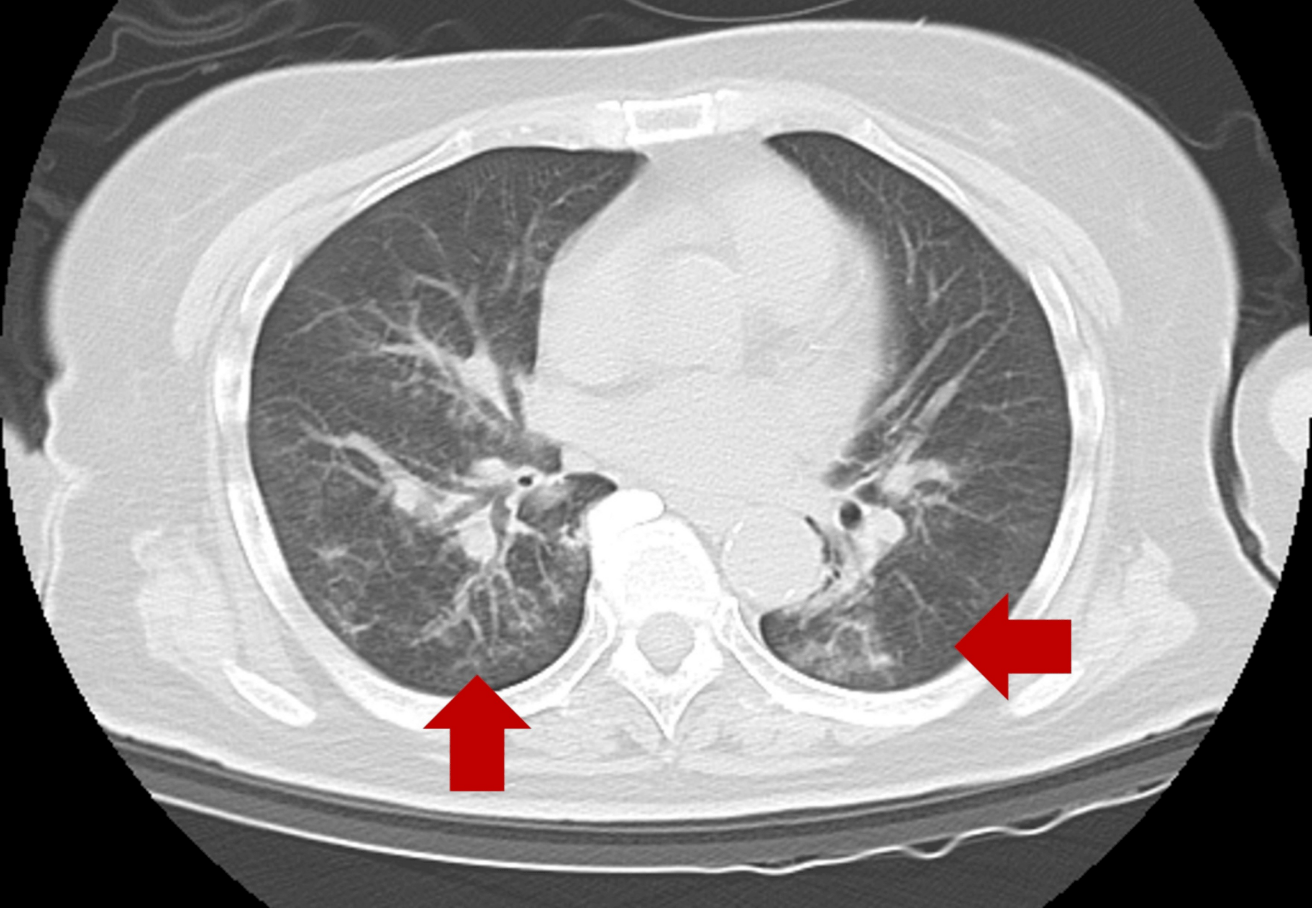
Chest Computed Tomography on Arrival The area indicated by the arrows demonstrates a pulmonary infiltrate consistent with pneumonia.

Laboratory testing revealed renal dysfunction, and ultrasonography demonstrated an inferior vena cava diameter of 3 mm with complete collapse, suggesting dehydration. Accordingly, extracellular fluid infusion was initiated at 100 mL/h. Given the patient’s respiratory failure, along with vomiting and computed tomographic evidence of pneumonia (Figure 2), aspiration pneumonia was suspected, and ceftriaxone 2 g/d was started intravenously. In addition, based on the patient’s history of lithium carbonate use, lithium intoxication was considered, and a serum lithium level was obtained (the result became available on day 4).

By the following day (day 1), adequate intravenous fluid therapy had resulted in improved renal function. However, urine output was severely depleted at 10 mL/h, and her level of consciousness further deteriorated, with a GCS of 2-2-1. Although the serum lithium level obtained the previous day had not yet become available, lithium toxicity was strongly suspected based on clinical symptoms and a history of lithium carbonate use, prompting initiation of CHD. Dialysis was performed with a blood flow rate of 80-120 mL/min, dialysate flow of 1,000 mL/h, and filtration rate of 1,000 mL/h. No ultrafiltration was applied.

By day 2, her consciousness improved substantially (GCS 3-4-6). Muscle strength and tremors gradually recovered, renal function improved with a creatinine level of 0.59 mg/dL, and spontaneous urination resumed (550 mL over eight hours).

On day 3, she was alert (GCS 4-4-6) and able to resume oral intake. Blood pressure was stabilized using nicardipine, and her respiratory and circulatory status remained stable. CHD was discontinued on day 5 after marked clinical and neurological recovery. At that point, vomiting had resolved, and she was transferred from the intensive care unit to a general medical ward. All psychiatric medications, including lithium, were discontinued.

Lithium carbonate was not resumed during the hospitalization. She experienced no recurrence of gastrointestinal symptoms or altered consciousness, and her psychiatric condition remained stable. She was transferred to a rehabilitation facility on day 11.

## Discussion

Lithium toxicity is a potentially life-threatening condition that manifests with neurological, gastrointestinal, and cardiovascular symptoms. The decision to initiate extracorporeal therapy depends on clinical presentation, renal function, and serum lithium levels. IHD remains the gold standard because of its rapid clearance rates (up to 100 mL/min) [7]. However, it may cause hemodynamic instability and post-dialysis rebound-an increase in serum lithium concentration after treatment due to redistribution from intracellular compartments [8].

In contrast, CHD or CRRT allows for gradual and continuous lithium removal, minimizing rebound while maintaining hemodynamic stability [5,9]. Prior studies have reported that continuous modalities achieve clearance rates comparable to IHD (~40-60 mL/min) and result in favorable outcomes even in elderly or critically ill patients [9]. Hybrid techniques such as sustained low-efficiency dialysis (SLED) have also shown effectiveness in preventing rebound toxicity while maintaining solute clearance.

In this case, the patient exhibited severe neurologic impairment, respiratory instability, and QT prolongation on electrocardiography, raising concern for potential hemodynamic deterioration. For these reasons, CHD was selected instead of IHD. Although the patient ultimately did not experience hemodynamic compromise, and IHD might have been feasible in retrospect, CHD resulted in steady improvement in consciousness and motor function without recurrence or hemodynamic complications. These findings are consistent with previous reports supporting CHD as a safe and effective alternative for lithium intoxication in hemodynamically unstable or elderly patients [9].

Real-time measurement of serum lithium concentrations is not available at our institution, and many hospitals face similar limitations. Therefore, management should not rely solely on serum lithium values. When lithium intoxication is suspected-based on neurologic and physical findings in conjunction with a history of lithium carbonate use-early initiation of IHD or CHD, tailored to the patient’s risk of hemodynamic instability, may optimize recovery and help prevent long-term sequelae, even before serum lithium concentrations become available.

## Conclusions

This case highlights the effectiveness and safety of CHD in the management of severe lithium intoxication, particularly in patients with neurologic impairment or potential hemodynamic instability. Although IHD remains the standard therapy, CHD offered gradual lithium clearance, prevention of rebound toxicity, and stable cardiovascular support, resulting in full neurological recovery in this elderly patient. Early initiation of extracorporeal therapy should be considered when lithium poisoning is suspected, even before serum lithium concentrations are available, especially in institutions where timely lithium measurement is not feasible. Clinicians should integrate clinical presentation, renal function, and patient stability when selecting the optimal dialysis modality to minimize morbidity and optimize outcomes.
